# Regulations of the Edinburgh College of Surgeons, Respecting the Qualifications of Candidates

**Published:** 1829-10-01

**Authors:** 


### REGULATIONS TO BE OBSERVED BY CANDIDATES FOR DIPLOMAS FROM THE ROYAL COLLEGE OF SURGEONS OF EDINBURGH.

THE present is decidedly the age of me-

---

dical legislation, every month sending out a new batch of laws from either English, Irish, or Scotch corporations. If wisdom is with laws, as Solomon said it was with counsellors, in their *multitude*, then decidedly we are the wisest medical community under the sun, for we really possess as many codes as all the rest of the world en masse. The mischief of it is, that the law of to-day is the dead letter of to-morrow, and the curricula of our colleges are perfect ephemerides, making a buz in a summer's sun but resolved into their constituent elements ere nightfall. We really wish our legislating gentlemen would erect something solid, either good or bad, for really a despotism is better than an anarchy. What a happy thing it would be for the nation, we mean of course the professional part of it, if a little of the *immutability* of the Medes and the Persians would infuse itself into the collective wisdom of Lincoln's-Inn Fields, or that some surgical Napoleon would arise, and promulgate a codex with some sort of stability. However, being neither revolutionists nor radicals we bear our *oppressions*, to use the cant term, with much good nature, and are disposed to be thankful for any good that may emanate from the powers that be.

The little pamphlet whose title precedes these observations has been sent us from Edinburgh, and deserves to be noticed in this Journal. After earnestly recommending the parents, guardians, and masters of young men about to enter the profession, to enjoin and enforce a good preparatory education in Latin, Greek, the modern languages, mathematics, and natural philosophy, the College proceed to lay down the following rules :—

"Schools of Medicine,—Courses of Lectures,—Qualification of Teachers, &c.

"Every Candidate for a Surgical Diploma must have followed his Studies in a University; or Established School of Medicine; or in a Provincial School specially recognised by the College, and which shall conform to such laws and regulations as have been, or may hereafter be enacted.

"Under the title Established School of Medicine, are comprehended all places in this country, where Diplomas in Surgery are granted, and such foreign Schools as are acknowledged by the constituted authorities of the countries where they exist.

"No Provincial School shall be recognised where there is not a General Hospital, containing at least eighty beds, at which regular Medical and Surgical attendance is given, *and where there are not established Courses of Lectures on Anatomy and Chemistry.*

"No Course of Lectures given at a Provincial School shall be recognised, unless it be of the same extent and duration as the Course required on the same subject in Edinburgh.

"The extent and period of study allowed to be gone through at a Provincial School, will be regulated by the means and facility of study which the College receive evidence of its affording; but, in all cases, at least two Winter, or one Winter and two Summer Sessions of the Course of Study required for a Diploma, must be passed at a University, or at one of the Established Schools of Medicine.

"In Edinburgh, the Lectures to be attended as part of the Surgical Curriculum shall be delivered by Professors in the University, or Fellows of the Royal Colleges of Physicians or Surgeons there: and elsewhere, by Professors of Universities, or by Fellows of the Royal College of Physicians of Edinburgh, and Fellows or Licentiates of the Royal Colleges of Physicians of London and Dublin, by Fellows of the Royal Colleges of Surgeons of Edinburgh, London, and Dublin, and of the Faculty of Physicians and Surgeons of Glasgow; and by persons holding a Medical Degree or Surgical Diploma, whose Courses of Lectures have been recognised by the College on special application.

"No tickets of a Professor or Lecturer shall be recognised, who teaches more than two of the branches required by the College; but Anatomy, with Practical Anatomy; and Chemistry, with Practical Chemistry, shall each, in reference to this Regulation, be considered as one branch.

"COURSE OF STUDY.

"Every Candidate for the Diploma of the Royal College, either previously to, or during his Medical education, must have received regular instructions in the *Elements of Mathematics;* and must have attended a course of *Mechanical Philosophy* of at least *three* months' duration, delivered by a Professor of that branch in a University, a Lecturer in a public Institution, or a Teacher specially recognised by the College.

"The Candidate must have attended the following separate and distinct Courses of Lectures during a period of at least *four* Winter Sessions, OR *three* Winter and *three* Summer Sessions, provided that in each Summer Session he shall have attended one or more of the Courses prescribed or recommended by the College, exclusive of Hospital attendance, and also provided that the Summer Courses of Lectures shall not commence till after the conclusion of the Winter Courses.

| | | Duration at least. |
|---|---|---|
| Anatomy, | 2 Courses, | Six Months each |
| Practical Anatomy, | 1 Course, or 2 Courses, | Six Months; Three Months each. |
| Chemistry, | 1 Course, | Six Months. |
| Practical Chemistry, The number of Pupils in each Class being limited to 25. | 1 Do. | Three Months. |
| Materia Medica and Pharmacy, | 1 Do. | Six Months. |
| Institutions of Medicine, OR Physiology, | 1 Do | Six Months. |
| Practice of Medicine, | 1 Do. | Six Months. |
| Clinical Medicine During the period of attendance at the Hospital where they are delivered. | 1 Do. or 2 Courses. | Six Months; Three Months each. |
| Principles and Practice of Surgery, | 2 Courses | Six Months. |
| Or, Do. and Military Surgery.* | 1 Course 1 Do, | Six Months. |
| Clinical Surgery During the period of attendance at the Hospital where they are delivered. | 1 Do. or 2 Courses | Six Months. Three Months each. |
| Midwifery, and Diseases of Women and Children. | 1 Course. | Three Months. |

* "The Course of Military Surgery must be delivered by a Professor of that branch in a University; or by a Lecturer, who, in addition to the other required qualifications, has served in the Medical Department of the Army or Navy; and the Course of Lectures must be of at least six months' duration, Lectures being delivered at least thrice per week."

"With the exception of the Courses of Clinical Medicine, Clinical Surgery, and Military Surgery, in which Lectures are not delivered daily, the six months' Courses, delivered in Edinburgh by Fellows of the College, or others, are understood to consist of five Lectures per week for a period of not less than five months.

"Two *London* courses of three months each, on any of the above subjects, shall be taken as equivalent to one six months' course.

"The Candidate must also have attended for *eighteen* months a Public General Hospital, containing at least eighty beds; OR for *twelve* months such a *Public General Hospital,* and *six* months a *Medical* or *Surgical Hospital,* or *Dispensary,* recognised by the College on special application.

"The Candidate shall be required, in addition to the Tickets or proof of entry to the different Classes, to produce *Certificates* of his having attended these Classes, from the respective Professors or Lecturers; and in the case of Practical Anatomy, the Certificate must express that the Candidate has been actually engaged in the dissection of the human body, under the personal superintendence of the Professor or Teacher, during the course of his attendance.

"The Candidate, at the commencement of his examination, will be required to translate into English some portion of a Latin author.

"The following Order of Study is recommended as a guide to the Student, though not absolutely enjoined.

"First Year, ANATOMY—CHEMISTRY—MECHANICAL PHILOSOPHY, if not previously attended.

"Second Year, ANATOMY—PRACTICAL ANATOMY—INSTITUTIONS OF MEDICINE or PHYSIOLOGY—SURGERY—MATERIA MEDICA and PHARMACY, either in this or the Third year.

"Third Year, PRACTICE OF PHYSIC—CLINICAL SURGERY—PRACTICAL CHEMISTRY—HOSPITAL.

"Fourth Year, SURGERY, or MILITARY SURGERY—MIDWIFERY and DISEASES of WOMEN and CHILDREN—CLINICAL MEDICINE—HOSPITAL.

"Besides the Courses of Lectures on the different Branches of Medicine required by the College, they strongly recommend to Students to avail themselves of the opportunities they may possess of attending Lectures on MEDICAL JURISPRUDENCE, BOTANY, NATURAL HISTORY, COMPARATIVE ANATOMY, and PATHOLOGICAL ANATOMY.

"The Duration and Course of Study to be required of Apprentices, whose indentures commenced before 1st January, 1823, are still regulated by the laws in force previously to that date: The course of study of Apprentices and others, who entered upon their indentures or their medical studies generally after that date, and before 1st August, 1829, will be determined by the rules in force within this period:—The present regulations as to *Endurance and Course of Study* shall take effect as to all who enter upon their medical education *after* the last mentioned date.

"The regulations as to *Lecturers, Teachers,* &c. &c. are to take effect *immediately*."*

We cannot but acknowledge the general good sense that marks the foregoing resolutions, although the keen eye of private interest may, perchance, detect some minor objectionable points. The time, thank God, is passed for ever, when endeavours to improve the education of the rising professional youth of these isles were met by a storm of invective, lies, and bitter denunciations, in short, with all the ichor of festering, cancerous malignity:—

Not urged by fear, nor through misguided sense—
Blind zeal and starving need had some pretence—
But for the good old cause that did excite
Th' original rebel's wiles, revenge and spite.

The proposers of measures need not now entertain any dread of the foul-mouthed ravings with which they were wont to be assailed; but they are, and must ever be, amenable to the temperate expression of public opinion. We perfectly agree in the propriety of refusing to recognize any provincial school in which anatomy is not regularly taught, for nothing can be more preposterous than accepting attendance on a county hospital, from a pupil who has never had the means of becoming systematically acquainted with anatomy. Surgery can neither be practised nor understood by a person ignorant of anatomy, for the former is essentially bottomed on the latter, and is quackery without it. We have seen enough of the evil effects of hospital attendance under such circumstances, to have implanted the conviction of its mischiefs deeply in our minds, whether it be the *popular* opinion or not. At the same time we cannot perceive the necessity for making chemistry a sine quâ non, and we think, on reflexion, that the framers of the curriculum will erase it from the list of essentials to a provincial school. Of course we do not say that chemistry should be neglected, far from it; but why should it

* The further particulars respecting the Registration, Examination, and Rate of Fees, are matters of local interest, which do not come within our province.—REV.

*only* be named with anatomy, as requisite to be taught in the provincial school. Surely it may be obtained during the "two Winter, or one Winter and two Summer Sessions" to be *subsequently* passed at a university or established school. We cannot understand the *gist* of the regulation. No candidate, we are told, can be tolerated, unless he has received regular instructions in the ELEMENTS OF MATHEMATICS. Now really, though mathematics are excellent things, we do conceive that an amputation may still be performed by a surgeon, albeit he is unable to square the circle or the hypothenuse! With all due deference to the Examiners, we venture to say that they are travelling out of their beat, when they *insist* on this or that item of education not strictly connected with the profession. Their object and their office is to examine who are capable of acting as SURGEONS, not SCHOOLMASTERS! A good general education, and mathematics as a part of it, is a very desirable thing, but though it may be strongly recommended, and enforced to a certain degree, there is a Rubicon beyond which a body in the situation of the College of Surgeons should hesitate to venture. It is to be remembered that a large number of those who will appear at its tribunal are destined to minister to the lower and the poorer classes, who will not, nor cannot, afford a remuneration for the acquisition of *accomplishments*. If the expense of medical education be carried beyond certain limits, many will despair of conquering the obstacles that the *res angusta domi* has planted in their path, and either abandon the profession altogether, or practise it in an unqualified and empirical manner. The poor will thus become the prey of quacks.

The remainder of the regulations appear to be very good, except that they are slightly tinctured with the taste for including every thing. We would say ourselves that some time might be judiciously clipped from the more extraneous branches, and added to that for the study of anatomy and the attendance on the medical and surgical lectures. As much time, for instance, is ordered to be devoted to chemistry as to the practice of physic, and half as much as to anatomy. These, however, though objections, are of a very venial cast, and the whole tenor of the present curriculum evinces a deep and sincere desire on the part of those who framed it, to promote the interests of science and the respectability of their successors. Such disinterested motives are praiseworthy in the extreme, and will ensure the cordial co-operation of every one who loves and honours his PROFESSION. If the details are not perfect, the defects can be easily remedied as occasions offer; but the principle being good, no one but a fool or a knave would cavil at trifles, or try to overturn the whole for a flaw in a part.

> The tamp'ring world is subject to this curse,
> To physic their disease into a worse.

